# Carotid artery resection and reconstruction with superficial femoral artery transplantation: a case report

**DOI:** 10.1186/1758-3284-1-19

**Published:** 2009-06-17

**Authors:** Yoann Pons, Elsa Ukkola-Pons, Philippe Clément, Bernard Baranger, Claude Conessa

**Affiliations:** 1 Head and Neck Surgery Department, Hôpital du Val de Grâce. 74, Boulevard de Port Royal, 75230 Paris, France; 2 Radiology Department, Hôpital du Val de Grâce. 74, Boulevard de Port Royal, 75230 Paris, France; 3 Vascular Surgery Department, Hôpital du Val de Grâce. 74, Boulevard de Port Royal, 75230 Paris, France

## Abstract

**Introduction:**

Managing advanced head and neck cancer is often a difficult task, particularly when massive invasion of the carotid artery is present. However, en bloc resection can be a curative procedure, and reconstruction of the carotid artery limits the risk for stroke. The aim of this study was to describe the interest, indication, potential risks, and methods by which we carried out resections as well as reconstructions of the carotid artery using superficial femoral artery transplantation.

**Subjects and Methods:**

We presented one case of en bloc resection of the carotid artery with reconstruction with superficial femoral artery transplantation.

**Results:**

Postoperative care was uneventful. The patient did not suffer from neurological deficiency. After three years of follow-up, the patient survived without any cancer recurrence.

**Conclusion:**

The occurrence of massive cancer invasion into the carotid artery should not be a contraindication for surgery. En bloc resection of the carotid artery with revascularization using the superficial femoral artery allows for appropriate control of the cancer, and carries an acceptable level of neurological risk.

## Background

The carotid artery (common and/or internal) is invaded in 5 to 10% of cervical lymph node metastasis of head and neck squamous cell carcinomas. [1] En bloc resection allows for better regional control of the disease, and the five-year disease-free survival rate is almost 22%.[2] Several vascular materials can be used for reconstruction of the carotid artery. Here, we present one case of extended neck dissection involving carotid artery resection and reconstruction via transplantation of the carotid artery with the superficial femoral artery. The aim of this study was to describe the interest, indication, potential risks, and methods by which we carried out this procedure.

## Case report

**Figure 1:**
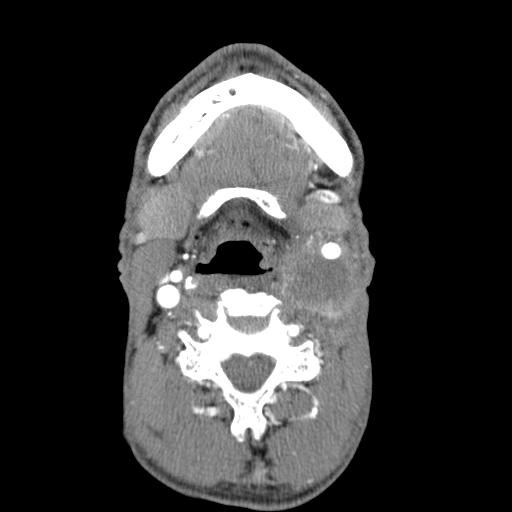
**Cervical CT-image (axial view, at the hyoid bone level) showing adenopathy invasion into the left common carotid artery**.

**Figure 2:**
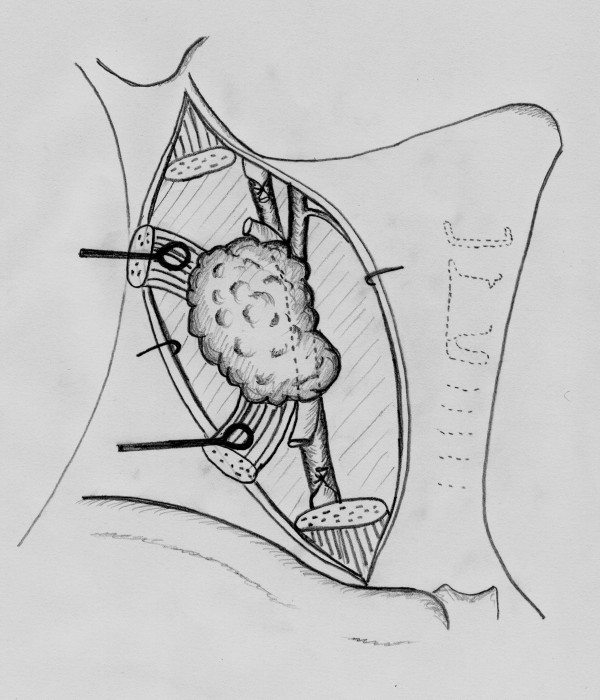
**Radical neck dissection**.

**Figure 3:**
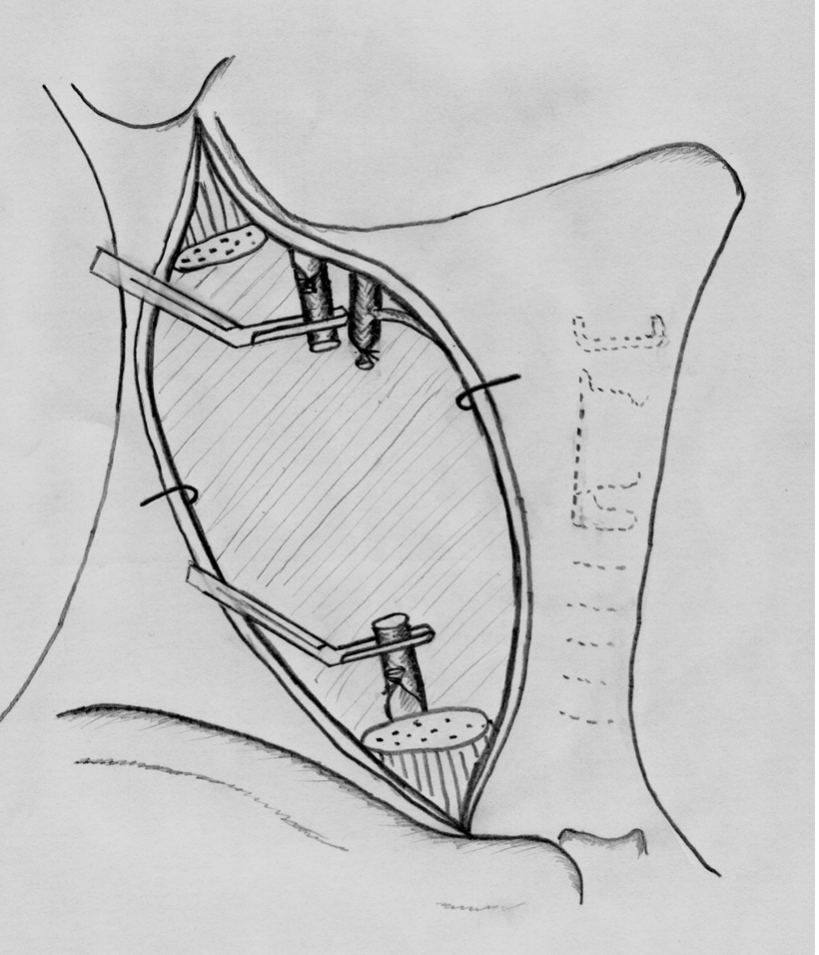
**The common and internal carotid arteries are clamped**. The external carotid artery is ligated.

**Figure 4:**
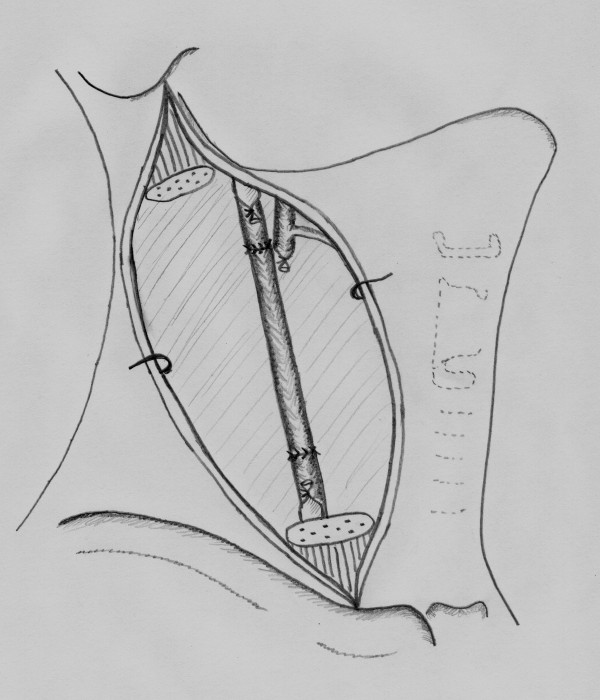
**Carotid artery reconstruction with the superficial femoral artery**.

**Figure 5:**
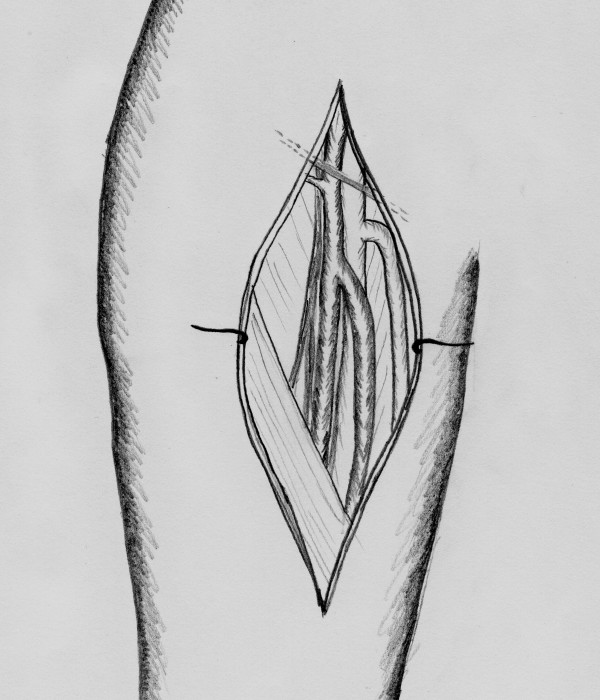
**The superficial femoral artery is located between the nerve and the vein**.

**Figure 6:**
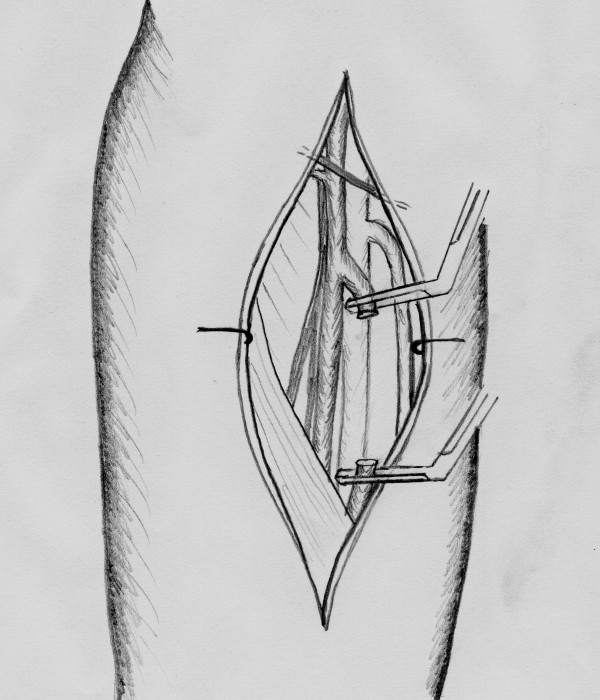
**The superficial femoral artery is clamped**.

**Figure 7:**
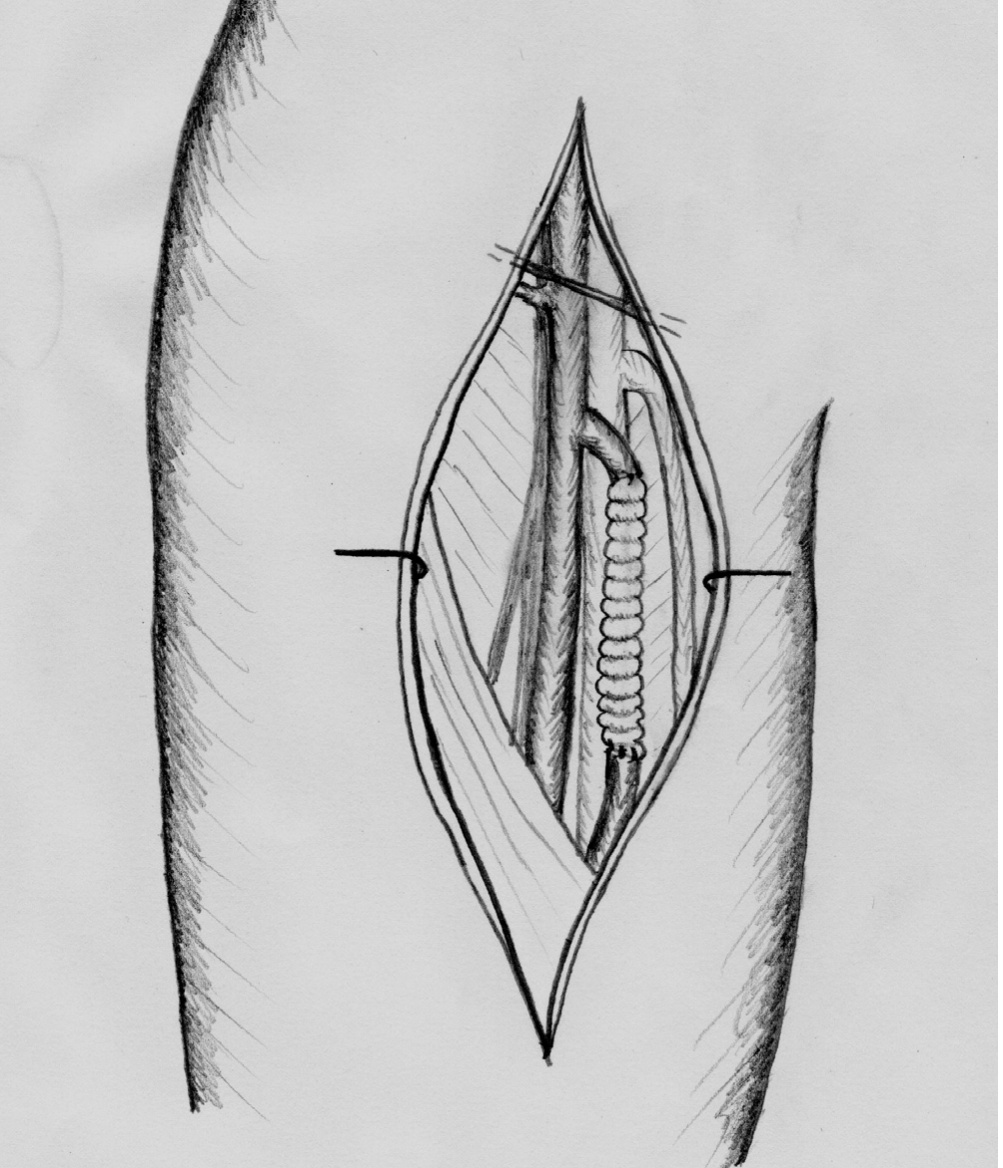
**The superficial femoral artery is reconstructed with a Dacron prothesis**.

**Figure 8:**
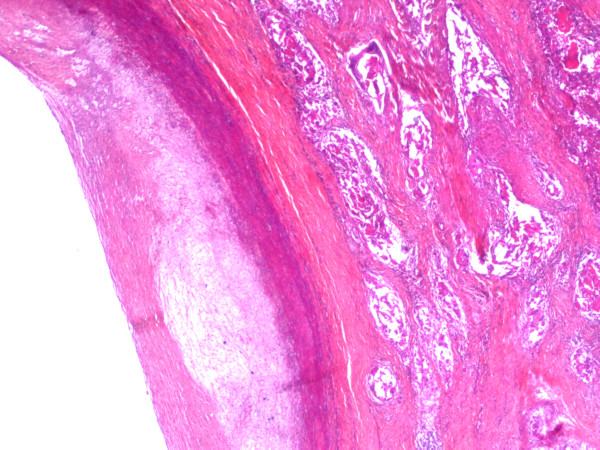
**Microscopic views (× 25) of the carotid adventitia invasion by the squamous cell carcinoma**.

**Figure 9:**
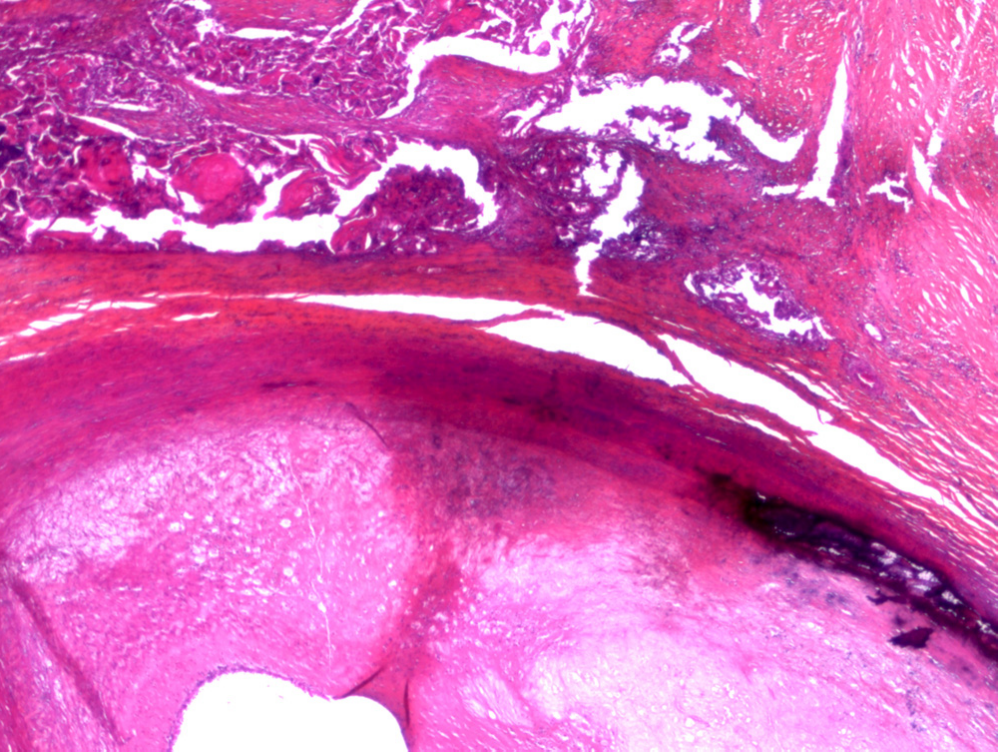
**Microscopic views (× 25) of the carotid adventitia invasion by the squamous cell carcinoma**.

**Figure 10:**
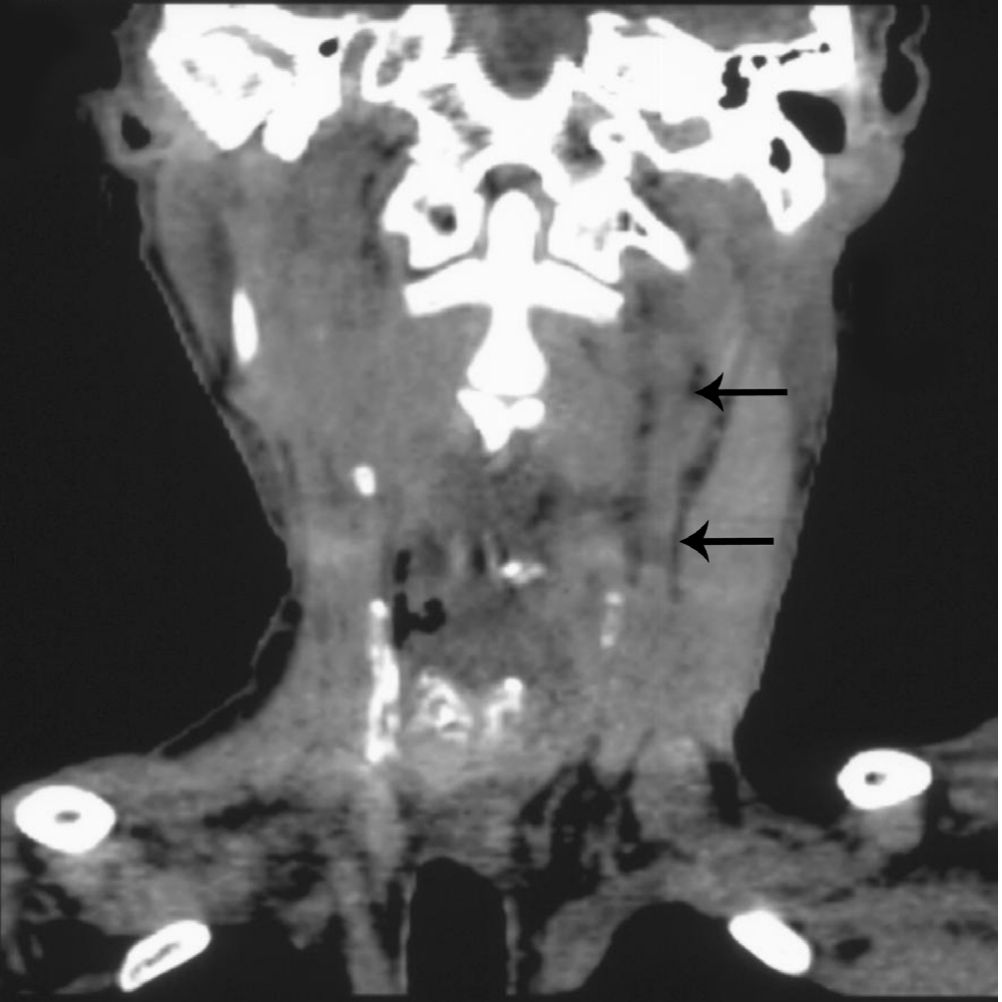
**CT frontal reformation of the neck extracted from the 18 FDG-PET/CT imaging**. The arrows show the reconstructed carotid artery.

**Figure 11:**
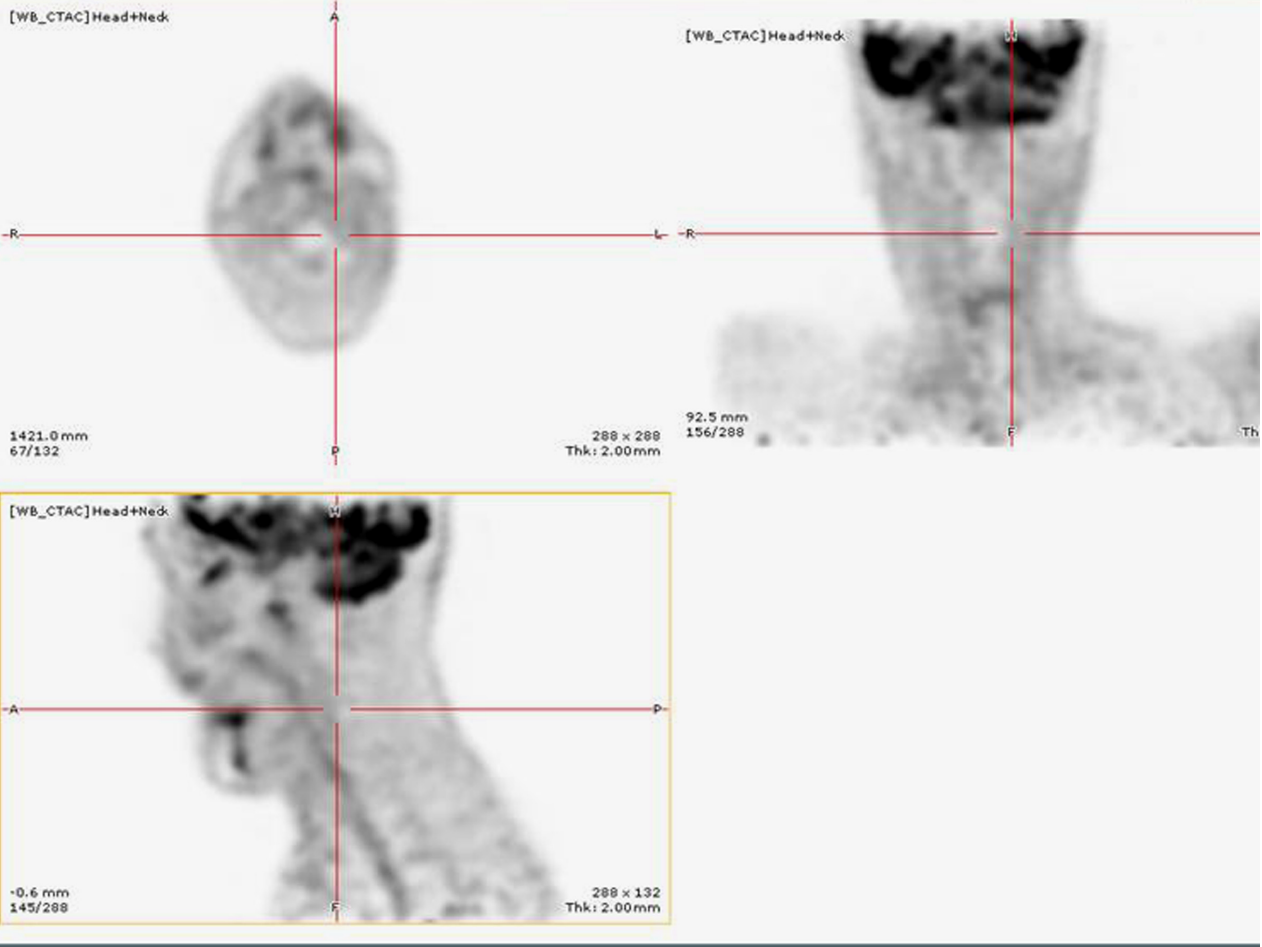
**Cervical 18 FDG extracted from the 18 FDG-PET/CT imaging showing no cervical recurrence of the cancer**.

A 52-year-old man was admitted to the head and neck surgery department for a left cervical adenopathy. No primary cancer site had been identified from endoscopic and imaging examinations. After three sessions of chemotherapy, both the scanner (figure 1) and MRI (figure 2) revealed an adenopathy that measured slightly more than 60 mm and exhibited invasion into the common and internal carotid artery. Angiographic examinations demonstrated a complete circle of Willis. We decided to carry out en bloc resection of the carotid artery and reconstruction with superficial femoral artery transplantation (figures 2, 3, 4, 5, 6 and 7). Clamping of the carotid artery lasted 25 minutes. Electroencephalogram (EEG) recordings during the operation did not elicit any cerebral pain. Post-operative care was uneventful, and the patient did not show any sign of neurological deficiency. The pathology analysis of the tumor confirmed that the carotid adventitia was invaded (figures 8 and 9). The patient benefited from cervical irradiation with a total administered dose of 70 grays. After three years of follow-up, the patient survived without any cancer recurrence, which was confirmed by 18 FDG-PET/CT (figures 10 and 11).

### Interest in en bloc resection of the carotid artery

En bloc resection of the carotid artery allows for better regional control of the cancer compared to other procedures, such as radio chemotherapy or carotid artery dissection only. [1] Nevertheless, the global survival rates at 5 years are identical for carotid artery dissection and en bloc resection. [2]

### Indications for en bloc resection of the carotid artery

When massive cancer invasion of the carotid artery is present (for instance, when the tumor invades the vessel over the adventitia), carotid artery dissection cannot be performed. Thus, in such cases, en bloc resection of the artery is necessary for proper surgical management of the cancer. Several imaging signs are useful for predicting a massive invasion of cancer into the carotid artery. These include deformation of the artery (which becomes oval), encasement of more than 180 degrees, and segmental obliteration of the fat between the adenopathy and the carotid artery. [3,4]

### Why do we systematically reconstruct the carotid artery?

The main risk associated with an en bloc resection of the carotid artery is cerebral stroke. If the carotid artery is removed but not reconstructed, the risk for developing a stroke is almost 30%. However, proper reconstruction of the carotid artery reduces this risk to less than 3%.[5]

### Pre-operative imaging

Arteriography, angio-TDM, or MRI allows one to verify the permeability of the Circle of Willis, and therefore minimizes neurological risk when performing arterial clamping. Arteriography is the gold standard and has the advantage of allowing one to carry out a clamping test.

### Technical aspects of a carotid artery resection and reconstruction via superficial femoral artery transplantation

Operative surveillance was conducted with EEG. However, similar to the use of evoked potentials, the efficiency of EEG in predicting neurological risk has not been fully demonstrated. Nevertheless, several operative assessments should be performed in order to prevent a stroke: maintenance of a stable median arterial pressure greater than 10 mmHg, achievement of a very short clamping time (less than 30 minutes if possible), and initiation of anti-coagulation (injection of 100 UI/kg of heparin) one minute before clamping. HHHeparin injection causes a more difficult tumor removal, and for this reason carotid reconstruction was performed last in our operative protocol.

### Possible materials for carotid reconstruction

The superficial femoral artery was used for the transplantation. The femoral artery has the advantage of having a comparable caliber to the common carotid artery, permitting good congruence in an arterial anastomosis. As a living tissue, the femoral artery is also resistant to infection following postoperative radiotherapy. In contrast, the absence of stenosis at the arterial level should be controlled by at least a Doppler echo. Moreover, transplantation of the femoral artery necessitates reconstruction of the section removed, which was replaced with Dacron prosthesis.

Certain authors use Dacron or ePTFE (expanded polytetrafluoretylene) prosthetics to carry out the carotid anastomosis. [5]

## Conclusion

The occurrence of massive cancer invasion into the carotid artery should not be a contra-indication for surgery. En bloc resection of the carotid artery with revascularization using the superficial femoral artery allows for appropriate control of the cancer, and carries an acceptable level of neurological risk.

## Abbreviations

CAD: carotid artery dissection.

## Consent

The patient gave their authorization for the publication of this case.

The Val of Grace's Review Board approved this study.

A copy of the written consent is available for review by the Editor-in-Chief of this journal.

## Competing interests

The authors declare that they have no competing interests.

## Authors' contributions

YP was the main redactor of the manuscript. EP helped him to redact the manuscript, particularly for the radiologic part of the text. PC helped to redact the manuscript. BB revised first the manuscript. CC defined the study design and performed the final revision of the manuscript.
